# Thermodynamic and Structural Study of Budesonide—Exogenous Lung Surfactant System

**DOI:** 10.3390/ijms25052990

**Published:** 2024-03-04

**Authors:** Atoosa Keshavarzi, Ali Asi Shirazi, Rastislav Korfanta, Nina Královič, Mária Klacsová, Juan Carlos Martínez, José Teixeira, Sophie Combet, Daniela Uhríková

**Affiliations:** 1 Department of Physical Chemistry of Drugs, Faculty of Pharmacy, Comenius University Bratislava, Odbojárov 10, 832 32 Bratislava, Slovakia; keshavarzi2@uniba.sk (A.K.); shirazi1@uniba.sk (A.A.S.); korfanta3@uniba.sk (R.K.); kanjakova2@uniba.sk (N.K.); klacsova@fpharm.uniba.sk (M.K.); 2 ALBA Synchrotron, Cerdanyola del Vallés, 08290 Barcelona, Spain; guilmar@cells.es; 3 Laboratoire Léon-Brillouin (LLB), UMR12 CEA, CNRS, Université Paris-Saclay, F-91191 Gif-sur-Yvette CEDEX, France; joseteixeira229@gmail.com (J.T.); sophie.combet@cea.fr (S.C.)

**Keywords:** lung surfactant, budesonide, differential scanning calorimetry, SAXS/WAXS, SANS, lateral pressure

## Abstract

The clinical benefits of using exogenous pulmonary surfactant (EPS) as a carrier of budesonide (BUD), a non-halogenated corticosteroid with a broad anti-inflammatory effect, have been established. Using various experimental techniques (differential scanning calorimetry DSC, small- and wide- angle X-ray scattering SAXS/WAXS, small- angle neutron scattering SANS, fluorescence spectroscopy, dynamic light scattering DLS, and zeta potential), we investigated the effect of BUD on the thermodynamics and structure of the clinically used EPS, Curosurf^®^. We show that BUD facilitates the Curosurf^®^ phase transition from the gel to the fluid state, resulting in a decrease in the temperature of the main phase transition (*Tm*) and enthalpy (Δ*H*). The morphology of the Curosurf^®^ dispersion is maintained for BUD < 10 wt% of the Curosurf^®^ mass; BUD slightly increases the repeat distance *d* of the fluid lamellar phase in multilamellar vesicles (MLVs) resulting from the thickening of the lipid bilayer. The bilayer thickening (~0.23 nm) was derived from SANS data. The presence of ~2 mmol/L of Ca^2+^ maintains the effect and structure of the MLVs. The changes in the lateral pressure of the Curosurf^®^ bilayer revealed that the intercalated BUD between the acyl chains of the surfactant’s lipid molecules resides deeper in the hydrophobic region when its content exceeds ~6 wt%. Our studies support the concept of a combined therapy utilising budesonide—enriched Curosurf^®^.

## 1. Introduction

Pulmonary surfactant (PS) is a surface-active complex of lipids (~90 wt%) and proteins that stabilise the air/liquid interface in alveoli [1,2]. It is produced, assembled, and secreted by type II pneumocytes in the form of lamellar bodies. These structures are then unravelled or directly absorbed into the air/liquid interface, forming a monolayer on the surface of the air/liquid interface [3,4]. To fulfil its functions, PS must follow the size of the alveoli, which means that it must be laterally compressed and then absorbed to the air/liquid interface and rapidly respread [5]. The chemical composition and presence of hydrophobic proteins (SP-B and SP-C), which are responsible for the stability of PS at the interface, facilitate the formation of 3D structures during the compression and expansion process [2]. 

Inactivation of PS refers to interference with its primary function, which is to reduce surface tension at the air/liquid interface. It eventually leads to bronchoalveolar collapse. Pathogenic molecules, generally not present in the alveolar space, can interfere with native PS and disrupt its function. For example, meconium aspiration syndrome (MAS) is a condition that occurs due to the aspiration of meconium-stained amniotic fluid into the lungs of the newborn [6,7]. Therapeutically, inactivated endogenous PS is replaced by exogenous surfactant (EPS) at a sufficient dose [8]. Intratracheal administration of EPS obtained from animals, e.g., porcine, Curosurf^®^ (Poractant Alfa); bovine, Survanta^®^, is currently a standard therapy in neonatal intensive care. Table S1 (Supplementary Materials) shows the composition of Curosurf^®^. 

Co-administration of EPS with corticosteroids has shown a higher efficacy in the treatment of the conditions mentioned. Budesonide (BUD) (Figure S1A, Supplementary Materials) is a non-halogenated synthetic steroid of the glucocorticoid family with a broad anti-inflammatory effect in different types of cells [9,10]. It is a non-polar molecule that is practically insoluble in water. The partition coefficient reported in the octanol/water system is Kp~1600 [11]. BUD was initially used as an inhaled treatment for inflammatory airway diseases. When administered in the form of an inhaler, BUD is associated with dysphonia and oropharyngeal *Candida* infections, as previously mentioned [12]. However, Decimo et al. [13] reported no evidence of dysphonia or oropharyngeal *Candida* infections with nebulised BUD. Intratracheal administration of BUD was found to be effective in suppressing meconium-induced inflammation [14]. The combination of porcine EPS and BUD is an effective therapy for pulmonary inflammation and oxidative modifications resulting from MAS [15,16]. Co-administration of BUD and EPS reduces lung injury markers and systemic responses [17]. In 2008, a pilot study of the intratracheal instillation of BUD-enriched EPS was conducted to prevent chronic lung disease in preterm infants, reporting a better lung outcome compared to the instillation of EPS alone [18]. Deliloglu et al. [19] suggested the combination of Curosurf^®^ enriched with BUD for the treatment of acute respiratory distress syndrome (ARDS) in newborns. 

How does the incorporation of BUD into EPS affect its biochemical and biophysical characteristics? The chemical stability of the BUD dispersed within the Curosurf^®^ was not affected during the 24-hour incubation at room temperature [20]. Biophysical studies have shown a drop (~30%) in the viscosity of the BUD/Curosurf^®^ mixture [20] and changes in the BUD/EPS mixture compared to EPS [20,21,22,23]. The functionality of EPS, to reach and sustain the necessary very low surface tension under compression–expansion cycles that mimic breathing dynamics, is not disrupted by the presence of BUD up to 10% by mass with respect to phospholipid content [5,23]. Furthermore, it was shown that the combination of clinical surfactant with corticosteroids (including BUD) efficiently promotes the active diffusion of the drug over long distances along the air/liquid interface [5]. Yeh et al. [18] confirmed that a large portion of BUD administered in combination with EPS remains in the lungs by AUC (total exposure of the drug over time) during the first 8 h after administration. Their finding is consistent with a report by Van Den Bosch et al. [24] stating that the concentration of BUD in lung tissue was eight times higher than its plasma concentration. To our knowledge, no considerable evidence of bioincompatibility has been reported for BUD dispersed in EPS. In general, all studies indicate good tolerance of EPS; however, cholesterol-free surfactant preparation appears to be more advantageous as a carrier of BUD [23].

Currently, trends are that EPS could be used as a carrier for pulmonary therapeutics [2]. For this purpose, it is important to assess the mutual drug—carrier interactions employing relevant experimental techniques. The functionality and structure of the pulmonary surfactant (both native and exogenous) are interconnected. For example, even ~1 wt% bacterial lipopolysaccharide (LPS) prevents Curosurf^®^ from reaching the necessary low surface tension during area compression in a dynamic system mimicking the respiratory cycle. Simultaneous research by a small-angle X-ray scattering experiment has shown that LPS disturbs the lamellar structure of Curosurf^®^ [25]. Therefore, we carefully selected experimental techniques for this study, to complete existing knowledge of BUD–EPS interactions, as briefly reviewed above. 

Here, we present results from structural studies employing techniques of X-ray and neutron scattering. Small-angle X-ray scattering (SAXS/WAXS) measurements revealed that the BUD slightly increases the repeat distance, *d*, of a lamellar phase of Curosurf^®^. A thickening of the lipid bilayer of Curosurf^®^ induced by BUD was derived from small-angle neutron scattering (SANS) experiments. Differential scanning calorimetry was used to investigate the fluidizing effect of BUD on Curosurf^®^ by means of determination of the gel-to-fluid phase transition *Tm*. The data obtained have shown the need for excimer fluorescence spectrometry to provide insight into the changes induced by BUD in the hydrophobic region of Curosurf^®^. In fact, excimer fluorescence is widely used to study lipid membrane systems. Numerous applications of the intramolecular pyrene-excimer formation technique have been initiated, among others, such as the determination of the gel–fluid phase transition [26], lamellar-non-lamellar phase transition [27], membrane fluidity [28], and monitoring of the lateral pressure within the bilayer [29]. All benefit from a long fluorescence decay lifetime (τ > 100 ns) and a high relative quantum efficiency (the intensity ratio of excimer emission to monomer emission) of bis-pyrenyl-phosphatidylcholine (Pyr*n*PC) fluorescence. Moreover, Pyr*n*PC emission spectra are highly dependent on temperature [30]. From a methodological point of view, further benefits of the technique may be listed, among them the time-efficient standardisation of procedures, providing results of good reproducibility and cost-effective approaches avoiding complex calibration steps. Further benefits include low sample concentrations needed and the applicability of the technique to a wide spectrum of membrane compositions and additives. We used excimer fluorescence spectroscopy to monitor changes in the lateral pressure at two depths of the hydrophobic part of the Curosurf^®^ lipid bilayer with the aim of elucidating the information on BUD localisation within the lipid bilayer of EPS.

## 2. Results

### 2.1. Thermodynamic Parameters

**Figure 1:**
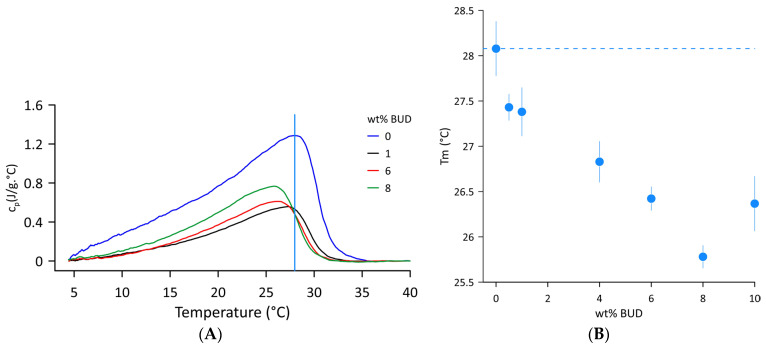
(**A**) DSC thermograms of the Curosurf^®^ and BUD/Curosurf^®^ mixture. The vertical line indicates the *Tm* of the Curosurf^®^. (**B**) Temperature Tm of the gel-to-fluid phase transition as a function of wt% BUD. The dashed line indicates the *Tm* of Curosurf^®^. The error bars represent the standard deviation of duplicate runs of the same sample.

Figure 1A shows the DSC thermograms of Curosurf^®^ and selected BUD/Curosurf^®^ mixtures recorded in the temperature range of 4–40 °C. A broad asymmetric endothermic peak signifies a complex phase transition from the gel to the fluid state with low cooperativity. The temperature *Tm* of the gel-to-fluid phase transition was derived from the position of the maximum, *Tm* = 28.1 ± 0.3 °C. The enthalpy Δ*H* = 18.0 ± 1.5 J/g and the phase transition width FWHM~12.5 °C were assessed from the values of the first and third scans (heating–cooling–heating regimen). The data obtained correspond well to the reported values [31,32,33]. The effect of BUD on the thermal behaviour of Curosurf® includes lowering the transition temperature (Tm) and decreasing the latent heat of the transition from gel to fluid. Figure 1B shows changes in *Tm* with increasing BUD content. The dashed line indicates the value of the *Tm* of Curosurf^®^. With increasing BUD content, the phase transition temperature decreases, reaching a value of 25.8 ± 0.1 °C for 8 wt% BUD. The enthalpy was reduced by more than half, with an average value Δ*H* = 9.13 ± 1.4 J/g in this range of BUD concentration. However, an additional increase in BUD content to 10 wt% results in a slight increase in *Tm* to 26.4 ± 0.1 °C and in enthalpy, Δ*H*~15.9 J/g. Individual DSC profiles are shown in Figures S2 and S3 and the parameters are summarised in Table S2 (Supplementary Materials).

### 2.2. Structural Study 

Morphologically, Curosurf^®^ is a mixture of unilamellar and multilamellar vesicles, as confirmed by freeze-fracture electron micrographs [34]. Figure S4 (Supplementary Materials) shows microscope images of aqueous dispersions of pure Curosurf^®^ and Curosurf^®^ in the presence of 4 wt% of BUD recorded in normal and polarised light. The microscopy was aimed at examining whether the fluidisation effect of the BUD could disintegrate the MLV ordering of Curosurf^®^. MLVs have anisotropic features due to the ordered concentric arrangement of their phospholipid layers [35,36], visualised under polarised light by polarisation crosses (also known as Maltese crosses). Maltese crosses are formed when light rays travel across an anisotropic material because of birefringence. The dark bands intersecting in the centre of the cross correspond to isotropic domains [37]. For pure Curosurf^®^, small clusters of vesicles were visible under normal light, which were recognised by Maltese crosses under polarised light as MLVs (Figure S4A,B), as previously reported [25,38]. BUD does not significantly perturb the multilamellar structure of Curosurf^®^; however, we observe fewer clusters of vesicles and Maltese crosses under normal and polarised light, respectively (Figure S4C,D). For comparison, a low concentration of Ca^2+^ (2 mmol/L) in the aqueous dispersion of Curosurf^®^ enhances the multilamellar structure due to the partial screening of its negative surface charge. The effect is evident for both the pure Curosurf^®^ and the BUD/Curosurf^®^ mixture with 4 wt% of BUD and Ca^2+^, as seen under normal and polarised light (Figure S4E–H).

### 2.3. SAXS/WAXS Study

**Figure 2:**
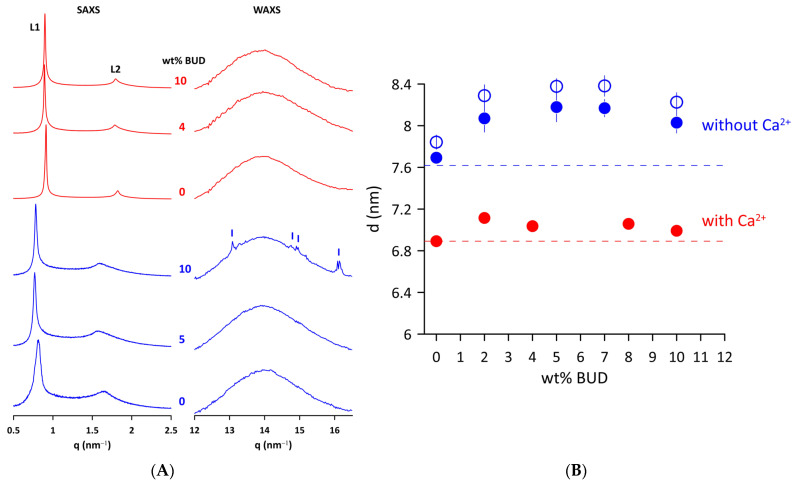
(**A**) SAXS and WAXS scattered intensity (in relative units) of Curosurf^®^ and BUD/Curosurf^®^ mixtures without (blue) and with 2 mmol/L of Ca^2+^ (red) at 50 °C. (**B**) Repeat distance (*d*) of Curosurf^®^ as a function of wt% BUD at 40 °C (empty blue symbols) and 50 °C (full blue symbols) in the absence or presence of Ca^2+^ (50 °C, full red symbols) in a hydration medium. The error bars are within the size of symbols. Dashed lines indicate the respective *d* of Curosurf^®^.

The effect of BUD on the structure of Curosurf^®^ MLVs in a fluid state was examined by X-ray scattering. Figure 2A shows the SAXS/WAXS patterns of the Curosurf^®^ and BUD/Curosurf^®^ mixtures without and with 2 mmol/L of Ca^2+^ at 50 °C. Two peaks (L1 and L2) of the SAXS pattern are characteristic of a lamellar phase formed by lipid bilayers of Curosurf^®^ arranged in an onion-like structure of MLVs. The repeat distance *d* = *d_L_ + d_W_* is the sum of the thickness of the lipid bilayer, *d_L_*, and the layer of water (*d_W_*) that separates the adjacent lipid bilayers. We found *d* = 7.8 ± 0.1 nm for Curosurf^®^ at 40 °C and a slightly lower value, *d* = 7.6 ± 0.1 nm at 50 °C. The electrostatic repulsion force between two neighbouring negatively charged bilayers (ξ~−7 mV) induces swelling of the onion-like structure (MLVs) of the Curosurf^®^. This, in turn, facilitates fluctuations in the lipid lamellae leading to broadening of the diffraction peaks [39,40], as can be seen in the SAXS patterns of Curosurf^®^ and BUD/Curosurf^®^. The addition of 2 mmol/L of Ca^2+^ to the lipid mixtures is enough to screen out electrostatic repulsions, leading to a better positional order of elementary cells and, finally, to narrow diffraction peaks (Figure 2, BUD/Curosurf^®^ in 2 mmol/L of Ca^2+^). We derived *d* = 6.9 ± 0.1 nm from the Curosurf^®^ SAXS pattern in the presence of Ca^2+^ at 50 °C. BUD does not substantially affect the long-range order of Curosurf^®^ MLVs; the patterns show only a minor thinning of the SAXS peaks. Figure 2B shows the repeat distance *d* derived from the SAXS patterns as a function of the content of the BUD (in wt%). We observe a small increase in *d* with the BUD content in both systems studied and at temperatures; however, this is less pronounced in the presence of Ca^2+^.

The right panel of Figure 2A displays WAXS patterns of Curosurf^®^ and BUD/Curosurf^®^ mixtures without and with 2 mmol/L of Ca^2+^. A broad peak at *q*~14 nm^−1^ is characteristic of liquid-like ordered lipid acyl chains [41]. Note that the WAXS pattern of Curosurf^®^ with 10 wt% BUD shows a few sharp small peaks (marked) superposed to a broad peak of liquid-like ordered lipid acyl chains. We identified peaks at *q* = 13.1, 14.8, 14.9, and 16.2 nm^−1^, respectively, which are consistent with peaks of the XRD pattern of the powder BUD reported in [42]. A similar effect was previously observed and reported, for example, for sitosterol in the egg yolk phosphatidylcholine bilayer [43] or long-chain alcohols in the mixture of dioleoylphosphatidylethanolamine (DOPE)/dioleoylphosphatidylcholine (DOPC) [44]. It should be noted that we did not observe a similar effect for BUD/Curosurf^®^ in the presence of Ca^2+^ (Figure 2A). 

### 2.4. SANS Study

**Figure 3:**
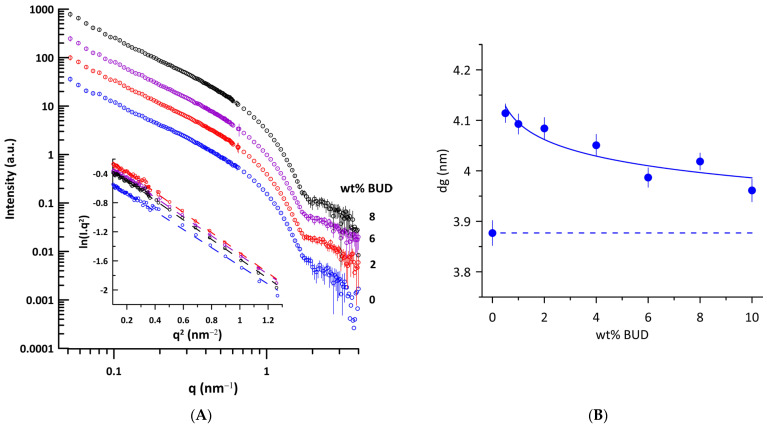
(**A**) SANS curves of unilamellar vesicles of Curosurf^®^ (blue empty circles) and BUD/Curosurf^®^ with different content of BUD (in wt%) at 40 °C. Curves are shifted along the y-axis for clarity. Inset: Kratky–Porod plot representation of scattering curves (*ln I*(*q*)*.q*^2^ vs. *q*^2^). (**B**) Effect of BUD on the thickness of the lipid bilayer *d_g_* of Curosurf^®^. The dashed line displays the value of *d_g_* of Curosurf^®^. The error bars were derived from the standard deviation of the slope of the Kratky–Porod plot.

We performed small-angle neutron scattering (SANS) experiments on ULVs of Curosurf^®^ and BUD/Curosurf^®^ mixtures to determine the thickness of the lipid bilayer, taking advantage of the contrast between the coherent neutron scattering length densities (NSLDs) of the Curosurf^®^ mixtures and of the solvent (D_2_O). Figure 3A shows the curves of SANS intensity *I*(*q*) vs. *q* typical of unilamellar vesicles prepared by extrusion. The parameter related to the thickness of the lipid bilayer, *d_g_*, was obtained from the value of the radius of gyration, *R_g_*, using Equations (3) and (4) (Materials and Methods). Additionally, *d_g_* is a linear function of the trans-bilayer phosphate–phosphate distance (*d_HH_*) in unilamellar phosphatidylcholine vesicles [45]. The Figure 3A inset shows the illustrative dependences of the Kratky–Porod plot, *ln*(*I*(*q*)*.q*^2^) vs. *q*^2^ in the region of 0.31 nm^−1^ ≤ *q* ≤ 1.14 nm^−1^ used for determination of *R_g_* (r^2^ ≥ 0.993). We determined the thickness of the lipid bilayer *d_g_* = 3.88 ± 0.02 nm for Curosurf^®^. For comparison, *d_g_* = 4.03 ± 0.11 nm was found for unilamellar DPPC vesicles at 50 °C, prepared by extrusion [46]. Figure 3B shows changes in the thickness of the lipid bilayer induced by BUD. Even 0.5 wt% of BUD increases the bilayer thickness by ~0.23 nm and, surprisingly, the effect decreases with the increasing BUD content.

### 2.5. Lateral Pressure

To gain insight into the partitioning of BUD in the Curosurf^®^ bilayer, we employed excimer fluorescence to monitor the changes in the lateral pressure across the bilayer of the BUD/Curosurf^®^ mixtures. In the phospholipid bilayer at the hydrophilic/hydrophobic region, the negative pressure prevents penetration of water into the hydrophobic core. This negative pressure, which is due to strong attractive forces between phospholipid glycerol groups, is compensated by the positive pressure imposed by two different forces. The first is repulsion between head groups due to steric, charge, and hydration effects and the second is repulsion between acyl chains due to entropic-driven collisions [29]. The overall lateral pressure across the lipid bilayer is zero. 

The excimer formation of single-pyrene-labelled phosphatidylcholines in the lipid membrane is a diffusion-controlled intermolecular collisional event occurring in the acyl chain region. Contrary to this, in bis-pyrenyl phosphatidylcholines (Pyr*n*PCs, where two fluorophores are attached to the same molecule, Figure S1B,C), excimer formation refers to an intramolecular collision event. The rate of this intramolecular excimer formation event depends on the lateral and intra-rotational dynamics and on the constraints imposed by the molecular environment of the probe. This enables evaluating a relative quantum yield efficiency (the intensity ratio of the excimer emission to the monomer emission), which was shown to be very sensitive to the bilayer structure, the presence of additives, and the temperature [47].

In the immediate vicinity of the probe, the lipid acyl chain structure was found to be strongly perturbed in the ordered gel phase, probably because of the bulky character of the pyrene moiety. However, no significant effect was observed on fluid-phase lipids [26]. In particular, all the samples studied in our work were under experimental conditions in a fluid phase. The rate of intramolecular excimer formation is, at sufficiently low probe levels (probe:lipid = 1:1500, as used in our study), independent of probe concentration [28]. The evaluated lateral pressure parameter (see Methods, Equation (5)), thus, simply reflects the probability that the two pyrene moieties attain the separation and relative orientation required for excimer formation, within the lifetime of the excited pyrene monomer. According to the above-mentioned, no direct interaction of the pyrene moiety with either of the components of the studied system is assumed. 

**Figure 4:**
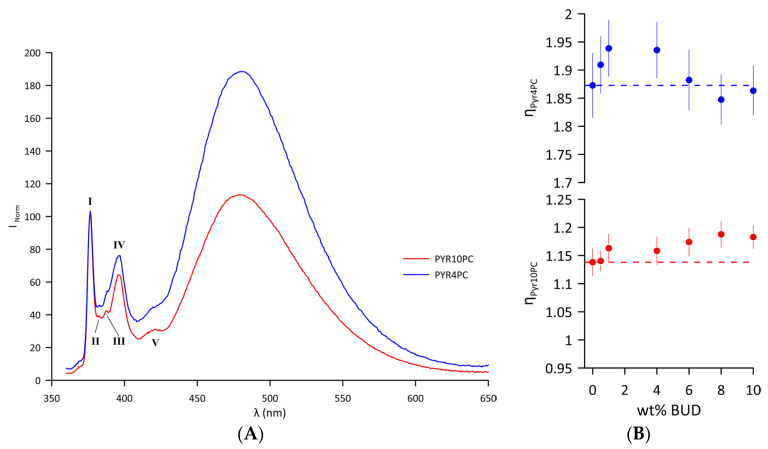
(**A**) Normalised fluorescence emission spectrum of Pyr4PC and Pyr10PC in Curosurf^®^ bilayers at 37 °C with designated vibronic bands of pyrene monomer (I–V). The spectra are normalised to the intensity of the first monomer maxima, $I_{monomer}^{norm}\left(376\right)=100$. (**B**) Lateral pressure detected by Pyr4PC (blue) and Pyr10PC (red) fluorescence probes as a function of wt% BUD at 37 °C. The dashed line corresponds to the *η_PyrnPC_ n* = 4 and 10 of Curosurf^®^ without BUD. The error bars represent the standard deviation of the fluorescence intensities measured as a function of time.

Changes in the lateral pressure of the Curosurf^®^ bilayer resulting from the inclusion of BUD were examined near the hydrophilic/hydrophobic interface and close to the hydrophobic bilayer core using two different fluorescence probes, Pyr4PC and Pyr10PC (Figure S1B,C), respectively. Emission spectra of the probes intercalated in the Curosurf^®^ bilayer (T = 37 °C) are displayed in Figure 4A. The first sharp peak at *λ_monomer_* = 376 nm belongs to the vibronic band I of the pyrene monomer. The interaction of two monomers forms an excited state dimer (excimer) with a broad, unstructured peak with a maximum at *λ_excimer_* = 480 nm. Excimer formation is enhanced when the average number of collisions per second increases as a result of increased lateral pressure. The normalised emission spectrum of both probes indicates that the lateral pressure is generally higher in the hydrophilic/hydrophobic interface compared to the hydrophobic core of the bilayer. For Curosurf^®^, the lateral pressure values at two depths of the hydrophobic region at 37 °C are *η_Pyr4PC_* = 1.87 ± 0.05 and *η_Pyr10PC_* = 1.13 ± 0.02. At 37 °C, a single lipid DOPC fluid membrane exhibited a significantly lower lateral pressure at the core of the lipid bilayer *η_Pyr10PC_* = 0.88 ± 0.02 compared to Curosurf^®^. However, the lateral pressure close to the hydrophilic/hydrophobic interface in DOPC approximates the values of pure Curosurf^®^ (*η_Pyr4PC_* =1.77 ± 0.05) [48]. Figure 4B summarises changes in the lateral pressure of the bilayer of the BUD/Curosurf^®^ mixtures. The BUD molecules intercalated in the lipid bilayer of Curosurf^®^ affected the lateral pressure differently: increasing the content of BUD leads to biphasic changes in the lateral pressure close to the hydrophilic/hydrophobic interface monitored by Pyr4PC (Figure 4B, blue symbols); meanwhile, it increases the lateral pressure in the hydrophobic core, as monitored by Pyr10PC (Figure 4B, red symbols). Both the values *η_Pyr4PC_* and *η_Pyr10PC_* indicate “a steady state” at a BUD content > 6 wt%.

## 3. Discussion

Curosurf^®^ consists of 99 wt% phospholipids and 1–2 wt% of hydrophobic surfactant proteins (SP-B and SP-C) [49]. Phosphatidylcholines represent 78 wt% and, among them, DPPC forms 35–56%. Negatively charged lipids (phosphatidylglycerol, phosphatidylinositol, phosphatidylserine) represent ~12 wt% [49]. The natural origin of Curosurf^®^ implies variation in its composition to some extent; however, the content of DPPC and the fraction of negatively charged lipids are crucial for the proper function of the surfactant. On the other hand, they determine the structure and thermodynamic behaviour of the surfactant. DPPC; the main surface-active component of PS [2,3] is composed of two saturated acyl chains (diC16:0 PC). When fully hydrated, there is a phase transition from a gel to a fluid state at *Tm* = 41.3 ± 1.8 °C [50]. Contrary to this, the melting phase transition from a gel to a fluid state of Curosurf^®^ (Figure 1A) differs from that of DPPC as it occurs over a large temperature interval. Such patterns are characteristic of complex lipid mixtures [51], forming domains with specific lipid compositions that melt at different temperatures [52]. In fact, the presence of double bonds in the lipid acyl chains results in a significant drop of *Tm*, for example, −2.5 ± 2.4 °C for POPC (16:0/18:1PC) or ~−18 °C for PLPC (16:0/18:2PC) [50]. The phase transition temperature *Tm* = 28.1 ± 0.3 °C and the enthalpy Δ*H* = 18.0 ± 1.5 J/g derived from DSC thermograms of Curosurf^®^ ULVs are in accordance with values of *Tm* = 27.3 ± 0.5 °C and the enthalpy Δ*H* = 21.1 ± 3.0 J/g determined for MLVs from five independent samples of Curosurf^®^ (different batches, not expired drug) [32] and the literature [31,33,52]. Differences in thermodynamic parameters (Tm, DH and the width, FWHM) were reported for ULVs and MLVs of saturated diacylphosphatidylcholines [50,53], in general, with lower DH detected for ULVs. 

Polarised light microscopy (Figure S4A,B) and SAXS identify predominantly multilamellar structures in Curosurf^®^. The repeat distance *d*~7.8 nm at 40 °C is less than previously reported *d*~8.4–9.4 nm [4,25,32], which we attribute to the natural origin of the surfactant. However, it is markedly higher than the *d* values~6.5–6.7 nm reported for phosphatidylcholines in the fluid L_α_ phase [39,54]. Based on the correlation of *dg*, *d_HH_*, and *d_L_* discussed in [45], we can consider that *d_L_* ≅ *dg* = 3.9 nm. This short analysis leads to the thickness of the water layer, *d_W_*~3.9 nm in Curosurf^®^ MLVs (at 40 °C), which is markedly higher compared to *d_W_*~1.8–2 nm found for fully hydrated phosphatidylcholines in the L_α_ phase [39]. The amount of water localised between neighbouring lipid bilayers in zwitterionic phosphatidylcholines is the result of a balance between repulsive interactions between bilayers (steric, hydration, and fluctuation) and attractive van der Waals forces [39]. The negatively charged lipids present in Curosurf^®^ induce electrostatic repulsion between the two neighbouring bilayers, resulting in a larger amount of water. The increase in temperature does not affect the repeat distance of Curosurf^®^ noticeably (Table S3); we found a slight decrease in *d*, *d* = 7.6 nm at 50 °C. Note that the presence of Ca^2+^, even at a concentration of 2 mmol/L (approximating physiological levels), affects MLVs arrangement of lipid bilayers of Curosurf^®^ (Figure S4E,F). Ca^2+^ ions screen the negative surface charge. We find *d*~6.9 nm and ~6.8 nm in the presence of 2 and 5 mmol/L of Ca^2+^, respectively, close to the *d* values reported for zwitterionic phosphatidylcholines. Ca^2+^, at these low concentrations, slightly increases the thickness *d_L_* of DPPC; however, the effect is negligible in unsaturated phosphatidylcholines [55,56]. Optimal adsorption of exogenous proteolipid extract surfactants requires the presence of Ca^2+^ in the subphase [57,58]. The physiological calcium concentration in the alveolus is ~1.5 mmol/L [59]. The surface tension function of commercial EPS was found to be strongly influenced by calcium; Curosurf^®^ showed a significant improvement once the Ca^2+^ concentration reached ~2–4 mmol/L [58]. On the other hand, molecular dynamics simulations show Ca^2+^—negatively charged phospholipid clustering [60,61] that can result in lateral phase separation. We did not detect any significant structural changes signalling impairment in Curosurf^®^, nor in BUD/Curosurf^®^ in the presence of a low Ca^2+^ concentration. 

### Effect of Budesonide

The DSC results show that BUD facilitates the phase transition of Curosurf^®^ from a gel to a fluid state: *Tm* decreases by ~2.4 °C with an increase in the content of BUD in the range 0.5 ≤ wt% of BUD ≤ 8 and a noticeable reduction in enthalpy is detected (Figure 1A,B). The fluidizing effect of BUD on Curosurf^®^ was previously reported [20,21,22,23] as being derived from BUD-induced changes in the Curosurf^®^ film. Our DSC measurements directly quantify the impact of BUD on the thermodynamic parameters of Curosurf^®^. Structurally, the BUD molecule is similar to cholesterol (CHOL). Both molecules feature three cyclohexanes and one cyclopentane in a fused ring structure, with different functional groups attached to this basic skeleton. In fact, a decrease in both *Tm* and Δ*H* was reported for binary mixtures of DPPC/CHOL [62,63,64] and, also, in more complex ternary systems [65]. However, in the case of Curosurf^®^, 10 wt% CHOL noticeably inhibits its surface properties due to the formation of a liquid-ordered phase [23].

The SAXS study confirmed that the multilamellar structure of Curosurf^®^ is preserved when BUD is incorporated into the lipid bilayer (Figure 2A,B), even if the population of MLVs appears to be lower in the micrographs taken under polarised light (Figure S4D). Repeat distance *d* increases non-linearly with the BUD content up to 8 wt% (Figure 2B); although, its change does not exceed ~0.5 nm. A slight drop in *d* at 10 wt% of BUD can be recognised in both studied systems (BUD/Curosurf^®^ without and with Ca^2+^). Simultaneously, the WAXS pattern of the BUD/Curosurf^®^ mixture with 10 wt% of BUD shows traces of BUD in the crystalline state (Figure 2A, WAXS). We hypothesise that at this high content, BUD is likely not fully “dissolved” in the hydrophobic region of the lipid bilayer of Curosurf^®^ (BUD: Curosurf^®^~1:5 mol/mol; supposing MW~762 g/mol for Curosurf^®^ [66]). The increase in thermodynamic parameters, *Tm* and Δ*H*, detected by DSC is in favour of this consideration. However, it is worth mentioning that sample preparation and, particularly, the history of samples, may play an important role. Note, that we did not observe a similar effect in the WAXS pattern of BUD/Curosurf^®^ with Ca^2+^ (Figure 2A, WAXS). 

In general, both the thickness of the lipid bilayer, *d_L_*, and also the layer of water, *d_W_*, can be affected by an additive in the lipid/water system. As BUD is practically insoluble in water, it is supposed to be completely incorporated into the Curosurf^®^ lipid bilayer, as confirmed by the electron spin resonance study [21]. Therefore, the observed increase in *d* with the BUD content (Figure 2B) reflects primarily the thickening of the lipid bilayer. The trend of both dependences, *d* vs. wt% of BUD, at 40 °C and 50 °C is similar, just shifted to lower values of *d* when the temperature increases. In the fluid L_α_ phase, an increase in temperature should induce an increased population of gauche rotamers in lipid acyl chains accompanied by a lateral expansion of the bilayer, resulting in an increase in area per molecule and a decrease in the steric lipid bilayer thickness, as observed, for example, for DPPC [54]. The system of BUD/Curosurf^®^ with Ca^2+^ (at 50 °C) shows a similar course *d* vs. wt% of BUD (Figure 2B). Indeed, the thickness of the lipid bilayer *d_g_* increases when BUD molecules are incorporated into the bilayer of Curosurf^®^. The maximal thickening, *d_g_*~+0.23 nm is caused by the addition of 0.05 wt% of BUD (BUD: Curosurf^®^~1:100 mol/mol). Further incorporation of BUD into the lipid bilayer of Curosurf^®^ results in a slight decrease in *d_g_*; however, thickening of the lipid bilayer is detected in the entire studied concentration range of BUD. The detected slight decrease is unusual and we hypothesise that it might result from non-uniform localisation of BUD in the hydrophobic region of the lipid bilayer of Curosurf^®^. There are obvious similarities between BUD and CHOL and their localisation in the lipid bilayer; however, much less is known about BUD. In fact, the increase in the thickness of the lipid bilayer due to CHOL embedded in ULV prepared from saturated and monounsaturated phosphatidylcholines using SANS and a similar approach as in this study was reported in our previous works [67,68]. The effect was more pronounced in saturated PC with short acyl chains (diC12:0PC) compared to those monounsaturated with long acyl chains (diC18:1PC or diC22:1PC). Recent findings support the concept of thickening of the lipid bilayer due to the CHOL-induced condensation effect [69] as dominant over the effect of the hydrophobic mismatch [70,71]. CHOL was found to be upright among phospholipid acyl chains, with its hydroxyl group located near the lipid–water interface. In this arrangement, CHOL modulates the structure of the lipid bilayer, leading to an increase in the thickness of the lipid bilayer, affecting the lateral area and the hydration of the phospholipid headgroup [70,72,73]. However, the affinity for CHOL decreases with the degree of unsaturation of the fatty acid chains that make up the lipid [74]. Other studies have shown that the orientation of the embedded CHOL molecule can be affected by the composition of the membrane, from the canonical upright found in lipid bilayers with saturated or monounsaturated acyl chains up to the “flat orientation” in the centre of the bilayer detected in polyunsaturated lipid membranes [75,76,77]. In summary, the lipid environment affects the location of CHOL in the hydrophobic part of the lipid bilayer.

The composition of Curosurf^®^ is complex, comprising lipid species with saturated, monounsaturated, and polyunsaturated acyl chains [4,78]. Therefore, the local hydrophobic environment might affect the BUD distribution in such a composite proteolipidic mixture. Excimer fluorescence measurements were made using two probes (Pyr4PC and Pyr10PC) in order to shed light on the localisation of BUD molecules within the lipid bilayer. Figure 4B summarises the changes in the lateral pressure. Even a few molecules of BUD induce an increase in lateral pressure close to the hydrophilic/hydrophobic interface, at the level monitored by Pyr4PC, resulting from the stiffening of the surrounding lipid acyl chains that, in turn, leads to the thickening of the lipid bilayer (detected by SANS). Note, the lateral pressure *η_Pyr4PC_* shows a breakpoint at ~4 wt% of BUD (BUD: Curosurf^®^~1:14 mol/mol), beyond which it decreases up to the level of *η_Pyr4PC_* detected for Curosurf^®^ itself. However, the lateral pressure in the vicinity of the hydrophobic core, monitored by Pyr10PC, gradually increases with the drug content up to ~8 wt% of BUD, indicating a denser packing of acyl chains in this region. It suggests either a change in the orientation of BUD molecules when the BUD content reaches BUD: Curosurf^®^~1:7 mol/mol or, more likely, their localisation deeper in the hydrophobic region. We do not observe notable changes in the lateral pressure when the content of BUD ≥ 8 wt%. These findings support the model of BUD molecules deposited deeper in the hydrophobic core when the drug content increases above 6 wt%. Simultaneously, such differences in the BUD localisation could result in a slight decrease in the thickness of the lipid bilayer, as shown in Figure 4B. The structural similarity of BUD molecules with CHOL and the results presented and discussed above support our hypothesis that BUD resides in the hydrophobic core of the bilayer, depending on the amount of the drug.

In summary, our study has confirmed a good tolerance of Curosurf^®^ for BUD up to < 10 wt% of the Curosurf^®^ mass. BUD facilitates the phase transition from a gel to a fluid state; however, its impact on the structure of EPS is minor. The lamellar structure is preserved and can be strengthened by the presence of Ca^2+^ at a low physiological concentration (~2 mmol/L). The BUD molecules are localised in the hydrophobic region, slightly increasing the thickness of the Curosurf^®^ lipid bilayer. Cholesterol is naturally present in the native lung surfactant and stimulates the formation of domains with liquid-ordered acyl chains. The experimental data obtained in this study do not allow us to detect domain-like organisation. Additional experimental methods are necessary to shed more light on the effect of BUD on the lateral ordering of bilayers. In this view, cholesterol-free formulations of EPS, such as Curosurf^®^, appear to be a more applicable delivery vehicle for BUD, to avoid the competitive binding of both structurally close sterols. Other studies have confirmed that the functionality of Curosurf^®^ enriched with BUD is preserved up to 10% by weight [5,23]. Deliloglu et al. [19], however, indicated that the practical and safe concentration of BUD administered in combination with Curosurf^®^ is 0.25 wt%. Contrary to Curosurf^®^, another EPS, Infasurf^®^ (calfactant), can only tolerate BUD up to 1% before losing its functionality [23]. Infasurf^®^ contains 5–8% cholesterol [23,79]. 

## 4. Materials and Methods

### 4.1. Chemicals

Curosurf^®^ (Poractant Alfa) was purchased from Chiesi Farmaceutici S.p.A (Parma, Italy). It is a modified natural surfactant, obtained from minced porcine lungs, reconstituted from almost exclusively polar lipids and hydrophobic proteins (SP-B and SP-C) without cholesterol. The composition of Curosurf^®^ is listed in Table S1 adapted from [49]. This off-white suspension has a phospholipid concentration of 80 mg/mL in NaCl solution and NaHCO_3_ is present for pH adjustments. Budesonide (BUD) was acquired from Sigma-Aldrich Chemie GmbH (Darmstadt, Germany). Excimer fluorescence probes (Pyr4PC, 1,2-bis-pyrene-butanoyl-phosphatidylcholine; and Pyr10PC, 1,2-bis-pyerene-decanoyl-phosphatidylcholine) (Figure S1B and Figure S1C, respectively) were obtained from Thermo Fisher Scientific (Waltham, MA, USA). Phosphorus pentoxide was purchased from Sigma-Aldrich Chemie GmbH (Darmstadt, Germany). Organic solvents (methanol and chloroform) of spectral purity were purchased from Slavus (Bratislava, Slovakia). CaCl_2_ solution (1.47 mg/mL) and NaCl stock hydration medium (9 g/L) were prepared by the dissolution of the required amount of CaCl_2_.2H_2_O (Sigma-Aldrich Chemie GmbH, Darmstadt, Germany) and sodium chloride (Lachema, Brno, Czech Republic) in ultrapure water (18.2 MΩ.cm, Labaqua Bio, Riga, Latvia), respectively. Deuterium oxide (isotopic purity 99.9% D_2_O; Merck, Darmstadt, Germany) was used as a solvent for the SANS measurement.

### 4.2. Sample Preparation

Curosurf^®^ suspension was dried over phosphorus pentoxide under a vacuum at ~7 °C. The dried lipid film was then dissolved in chloroform/methanol = 1:1 (*v*/*v*). The required amounts of Curosurf^®^ and BUD dissolved in organic solvents were mixed. For fluorescence measurements, Pyr*n*PC fluorescent probes (*n* = 4 and 10) dissolved in methanol were added in a Pyr*n*PC: (lipid + BUD) = 1:1500 molar ratio (the relative molar mass 762 was used for Curosurf^®^). The organic solvent was removed under a stream of nitrogen gas and the remaining residues of organic solvents were removed under a vacuum. The dried lipid films were rehydrated to maintain constant ionic strength. A small amount of concentrated CaCl_2_ solution was added to selected samples to reach its final concentration of 2 mmol/L (for DSC, SAXS/WAXS, and light microscopy). Samples were homogenised by vigorous vortexing and five freeze/thawing cycles (−50 °C/+50 °C). For excimer fluorescence, samples were briefly sonicated in an ultrasound bath (~45 °C). The obtained multilamellar vesicles (MLVs) were examined by light microscopy, excimer fluorescence, and SAXS/WAXS. The amount of BUD in the BUD/Curosurf^®^ mixture is reported in weight percent (wt%) with respect to the Curosurf^®^ mass.

For DSC and SANS, unilamellar vesicles (ULVs) were prepared by extrusion of the hydrated lipid dispersion through a 100 nm pore-size polycarbonate filter (Nuclepore, Pleasanton, CA, USA) using the Lipofast Basic Extruder (Avestin, Ottawa, ON, Canada), as described in [80]. Samples were subjected to 51 passes through the filter at a temperature of ~45–50 °C. The resulting solution showed only a marginal opalescence, which is typical for the dispersion of ULVs. Dynamic light scattering (DLS) has shown ULVs of diameter 79 ± 20 nm with a polydispersity of ~16% at a temperature of 20 °C (Figures S5 and S6). The zeta potential ζ = −6.9 ± 3.8 mV measured by electrophoretic light scattering at a temperature of 20 °C shows a negative surface charge of ULVs unaffected by the BUD content (Figure S7). Figure S8 shows the zeta potential as a function of temperature. No damage to the vesicles was detected during measurements. A schematic overview of sample preparation is depicted in Scheme S1, Supplementary Materials.

### 4.3. Methods

#### 4.3.1. Differential Scanning Calorimetry (DSC)

The effect of BUD on the thermotropic gel-to-fluid phase transition of Curosurf^®^ was studied using the Nano DSC calorimeter (TA instruments, New Castle, DE, USA). ULVs with a lipid concentration of 3 mg/mL were used for the measurements. All samples rested in the fridge overnight (~7 °C) before measurement. To avoid the formation of air bubbles during heating and cooling, before measurement, each sample was degassed at 0.329 atm for 15 min using a degassing station (TA instrument, New Castle, DE USA) at 4 °C. The measurement was performed without applying pressure and three cycles (heating—cooling—heating) in the temperature range of 2–40 °C with a heating rate of 1 °C/min^−1^ were carried out for each sample. Data were treated using OriginPro software (Version 2022, OriginLab Corp., Northampton, MA, USA). Calorimetric enthalpies (Δ*H*) were analysed using a standard integration procedure of areas under the peak after correction of the baseline and normalisation to the mass of the sample. The temperature of the main phase transition (*Tm*) was derived from the maximum of the peak. The reported values represent the mean *Tm* and Δ*H* of the first and third scans.

#### 4.3.2. Optical and Polarised Light Microscopy

Optical and polarised light microscopic studies were performed with a polarised light Nikon Eclipse LV100N POL microscope at laboratory temperature (~20 °C). Photographic images were recorded with a CCD Nikon DS-Fi2 camera (Tokyo, Japan) and NIS-Element Viewer software v. 4.2.

#### 4.3.3. X-ray Scattering (SAXS and WAXS)

Structural changes of the BUD/Curosurf^®^ mixture were examined by small- and wide-angle X-ray scattering (SAXS and WAXS). MLVs with a lipid concentration of 16 mg/mL were prepared for the measurements. Before measurements, each sample was centrifuged at 13,000 rpm for 2 min. The sediment of the lipid was transferred to a thin-walled borosilicate glass capillary (WJM-Glas Müller GmbH, Berlin, Germany) with a diameter of 1.5 mm and sealed to prevent evaporation. SAXS/WAXS experiments were performed at the BL11-NCD-SWEET beamline at the ALBA synchrotron (Barcelona, Spain), using linearly polarised radiation with a wavelength *λ* = 0.12 nm. The capillary was placed vertically in a Linkam heating stage, incubated for 5 min at the selected temperature, and exposed for 1 s to radiation. The SAXS and WAXS data were detected on a Pilatus 3S 1M detector (calibrated using silver behenate [81]) and an LX255HS Rayonix detector (calibrated using Cr_2_O_3_), respectively, (Norderstedt, Germany). The PyFAI python library was used to integrate azimuthally 2D scattering patterns data into 1D data [82]. The diffraction peaks were fitted with the Lorentzian function with a linear background. The repeat distance of the bilayer (*d*) was calculated as:

*d* = 2π/*q* (1)

where *q* is the position of the first structural peak.

#### 4.3.4. Small-Angle Neutron Scattering (SANS) 

The effect of BUD on the lipid bilayer thickness of Curosurf^®^ was examined by small-angle neutron scattering (SANS). ULVs prepared in heavy water (D_2_O) at a lipid concentration of 10 mg/mL filled in 2 mm thick quartz cells (Hellma, Müllheim, Germany) were used for the measurements. 

Neutron scattering experiments were performed on the PAXY spectrometer of the Orphée reactor (Laboratoire Léon Brillouin, Saclay, France). The scattered intensity, *I*(*q*), is measured as a function of the momentum transfer, *q*, which depends on the wavelength, *λ*, of the incident neutron beam and the scattering angle *2θ*; *q = 4π.sin*(*θ*)/*λ*. Two setups were used for the measurement: with sample-to-detector distances (S-D) of 1 m and 5 m and neutron wavelengths 0.5 nm and 0.85 nm, respectively, covering the domain of *q* = 0.05–5 nm^−1^ (resolution Δ*λ*/*λ* = 10%). The acquisition time for each sample was 20 min at S-D 1 m and 30 min at S-D 5 m. The samples were measured at a temperature of 40 ± 0.1 °C. Normalised SANS intensity, *I*(*q*), was obtained using the software Pasinet v.2.5. provided by the LLB. The spectra were corrected for the incoherent background. Because the coherent part of the intensity scattered at small angles vanishes for large values of *q*, we subtracted the measured constant value, which is mainly due to incoherent scattering (although including, as well, residual noise contribution).

Generally, for a system of monodisperse centrosymmetric particles, the coherent scattering intensity is given by:

*I*(*q*) = *N*·*P*(*q*)·*S*(*q*) (2)

where *N* is the number density of particles, *P*(*q*) is their form factor, and *S*(*q*) is the interparticle structure factor. *S*(*q*) ≅ 1 for dilute particles and those weakly interacting, which is a good approximation for ULVs prepared by extrusion at phospholipid concentrations < 2 wt% [83,84]. In the first approximation, unilamellar vesicles are hollow spheres with a lipid bilayer shell separating the inside and outside aqueous compartments. For such particles, the factor *P*(*q*) can be calculated by the one-dimensional Fourier integral of the coherent neutron scattering length density. According to the Guinier approximation, at small *q* (*q.R_g_* < 1) [85,86], and assuming that *S*(*q*) = 1, Equation (2) can be written as:

*I*(*q*) *= C exp* (*−q*^2^·*R_g_*^2^)·*q*^−2^ (3)

where *R_g_* is the one-dimensional radius of gyration of extended thin sheets and *C* is a constant. It is well known [85] that the thickness of the two-dimensional planar sheet *d_g_* can be obtained from the radius of gyration *R_g_* as:

*d_g_*^2^ ≅ 12 *R_g_*^2^ (4)

where *d_g_* is approximately equal to the thickness of the lipid bilayer in ULVs, supposing that the size of the vesicles is large compared to 1/*q* and that there is no water penetration inside the polar region of the bilayer [45,87]. The values of *R_g_* were obtained from the Kratky–Porod plot, *ln*(*I*(*q*)*·q^2^*) vs. *q^2^* in the region of 0.31 nm^−1^≤ *q* ≤ 1.14 nm^−1^.

#### 4.3.5. Excimer Fluorescence

Phosphatidylcholine-based excimer fluorescence probes the pyrene moieties located on the fourth carbon (Pyr4PC) and on the tenth carbon (Pyr10PC) of the acyl chain were used to monitor changes in the lateral pressure of the Curosurf^®^ at two depths of the hydrophobic region. These probes mix randomly in fluid and gel bilayers and are able to mimic the physical properties of non-fluorescent PCs in the bilayer [88,89]. The final concentration of lipid or lipid + BUD was 0.075 mg/mL for fluorescent measurements. The prepared samples rested in the fridge overnight. Before the measurement, samples were warmed up in the water bath, briefly sonicated, and vortexed [90]. Measurements were performed on a FluoroMax-4 fluorimeter (HORIBA Jobin Yvon, Longjumeau Cedex, France) using a quartz cuvette with a path length of 1 cm. The excitation wavelength *λ_ex_* = 345 nm was used to collect the emission spectrum in the region of *λ_em_* = 360–650 nm with an increment of 0.5 nm. The excitation and emission bandwidths were 5 and 2 nm, respectively. The fluctuation of the emission intensity was recorded during 120 s at *λ_em_* = 376 nm (pyrene monomer emission) and *λ_em_* = 480 nm (pyrene excimer emission) [29] (an illustrative pattern is shown in Figure S9). Measurements were performed at 37 ± 0.1 °C, maintained by a Peltier thermocouple drive. Samples were continuously stirred during measurement. Spectral responses were corrected by monitoring S1c/R1c values, where S1c and R1c indicate the signal detector correction and the lamp detector correction, respectively. The parameter of lateral pressure *η* within the lipid bilayer was calculated as the ratio of the excimer intensity to the monomer intensity at 480 and 376 nm, respectively [91].

*η* = *I_excimer_*/*I_monomer_* (5)

where *I_excimer_* and *I_monomer_* denote the average values of excimer and monomer emission intensity, calculated from the time-based measurement.

## 5. Conclusions

The benefit of combined therapy using an exogenous lung surfactant as a delivery vehicle for an additional drug is supported by scientific evidence. However, it requires careful inspection of the mutual interactions between the exogenous lung surfactant and the transported drug. In fact, exogenous pulmonary surfactant enriched with BUD has been clinically tested and there is evidence for tolerance of exogenous lung surfactant to BUD. Previous studies have focused on the functionality of the BUD/exogenous lung surfactant mixture and the ability to maintain the necessary surface-active properties as an essential role of the surfactant. Our studies contribute to the recent knowledge, focussing on the effect of BUD on the thermodynamics and structural changes of the porcine exogenous surfactant, Curosurf^®^. We have shown that BUD facilitates the phase transition from a gel to a fluid state of Curosurf^®^: it decreases the temperature of the main phase transition and noticeably reduces the necessary excess of heat (enthalpy, Δ*H*) in liposomal formulation. Structural studies show that the morphology of the Curosurf^®^ dispersion is maintained; BUD slightly increases the repeat distance of the lamellar phase in onion-like structures (MLVs), resulting from the thickening of the lipid bilayer. The presence of a low concentration of Ca^2+^ (~2 mmol/L, approximating physiological level) maintains the structure of the MLVs. The localisation of BUD in the lipid bilayer is discussed and supported by measurements of the lateral pressure at two depths of the hydrophobic region of the bilayer by using the excimer fluorescence technique. The results obtained allowed for insight into the interactions between BUD and a complex proteolipidic mixture, such as Curosurf^®^, and may be useful in the development of additional lipid-based carriers for corticosteroids. On the other hand, we underscore the necessity to employ additional techniques, for example, to shed more light on the lateral ordering and formation of domains. So far, our studies have contributed to recent knowledge and did not find any results discouraging the concept of a combined treatment with Curosurf^®^ enriched with budesonide. 

## Data Availability

The original contributions presented in the study are included in the article/Supplementary Materials, further inquiries can be directed to the corresponding author.

## Supplementary Materials

The following supporting information can be downloaded at: https://www.mdpi.com/article/10.3390/ijms25052990/s1.
