# Supplementary material for: Thermodynamic and Structural Study of Budesonide—Exogenous Lung Surfactant System
Source: Int J Mol Sci. 2024 Mar 4;25(5):2990. doi: 10.3390/ijms25052990 (PMC10931555; doi:10.3390/ijms25052990)
Supplement: Supplementary file 1 [file ijms-25-02990-s001.zip › ijms-2856873-supplementary.pdf]

# SUPPLEMENTARY MATERIAL

# Thermodynamic and structural study of budesonide - exogenous lung surfactant system

**Atoosa Keshavarzi[1], Ali Asi Shirazi[1], Rastislav Korfanta[1], Nina Královič[1], Mária Klacsová[1], Juan Carlos Martínez[2], José Teixeira[3], Sophie Combet[3], Daniela Uhríková[1#]**

*[1]Department of Physical Chemistry of Drugs, Faculty of Pharmacy, Comenius University Bratislava, Odbojárov 10, 832 32 Bratislava, Slovakia*

*[2]ALBA Synchrotron, Cerdanyola del Vallés, 08290, Barcelona, Spain*

*[3]Laboratoire Léon-Brillouin (LLB), UMR12 CEA, CNRS, Université Paris-Saclay, F-91191 Gif-sur-Yvette CEDEX, France*

[#]Corresponding author, e-mail: uhrikova@fpharm.uniba.sk

ORCID: Daniela Uhríková 0000-0002-4397-1283

**Figure S1** Chemical formulae of chemicals used for sample preparation:

A) Budesonide (BUD)

B) 1,2-bis-pyrene-butanoyl-phosphatidylcholine (Pyr4PC)

C) 1,2-bis-pyerene-decanoyl-phosphatidylcholine (Pyr10PC)

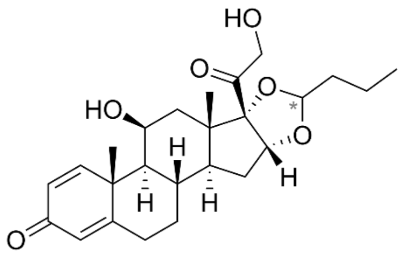

**A) BUD**

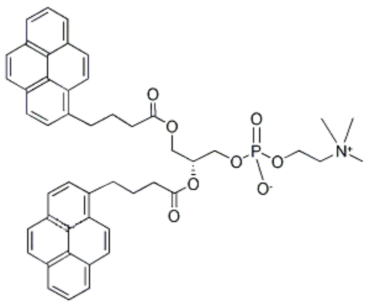

**B) Pyr4PC**

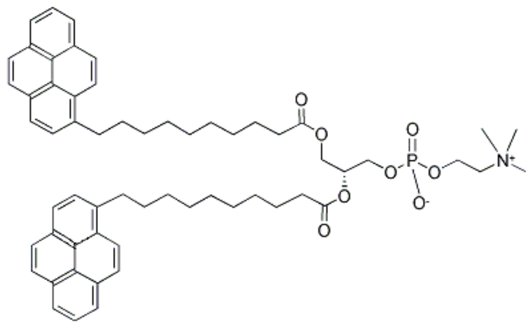

**C) Pyr10PC**

**Table S1** The composition of native pulmonary surfactant and clinically used exogenous pulmonary surfactant (EPS) Curosurf®

| | **Native Surfactant** | **Curosurf®** |
|---|---|---|
| PL | 80 - 90 | 99 |
| PC % (w/w) | 70 - 85 | 78 (67.5) |
| DPPC (%PC) | 36 - 54 | 35 - 56 |
| PE | 3 | 4.5 - 7.5 |
| PI | 4 - 7 | 3.3 - 7.2 |
| PG | 7 - 10 | 1.2 - 3.5 |
| PS | 5 | 1.2 ± 1.1 |
| SM | 2 | 1.8 ± 0.3-8 |
| LPC | 0.2 | < 1 - 6.9 |
| Chol | 5 | 0 |
| SP-B * | 10 - 11 | 2 - 3.7 |
| SP-C* | 22 - 34 | 5 - 11.6 |

Data presented in the table are in wt% with respect to the total mass of surfactant.
* Data expressed in µg protein/µmol of PL
PL: polar lipids PC: phosphatidylcholine; DPPC: dipalmitoylphosphatidylcholine; PE: phosphatidylethanolamine; PI: phosphatidylinositol; PG: phosphatidylglycerol; PS: phosphatidylserine; SM: sphingomyelin; LPC: lysophosphatidylcholine; Chol: cholesterol; SP-B, SP-C: surfactant specific protein B and C, respectively.
Table was adapted from [49].

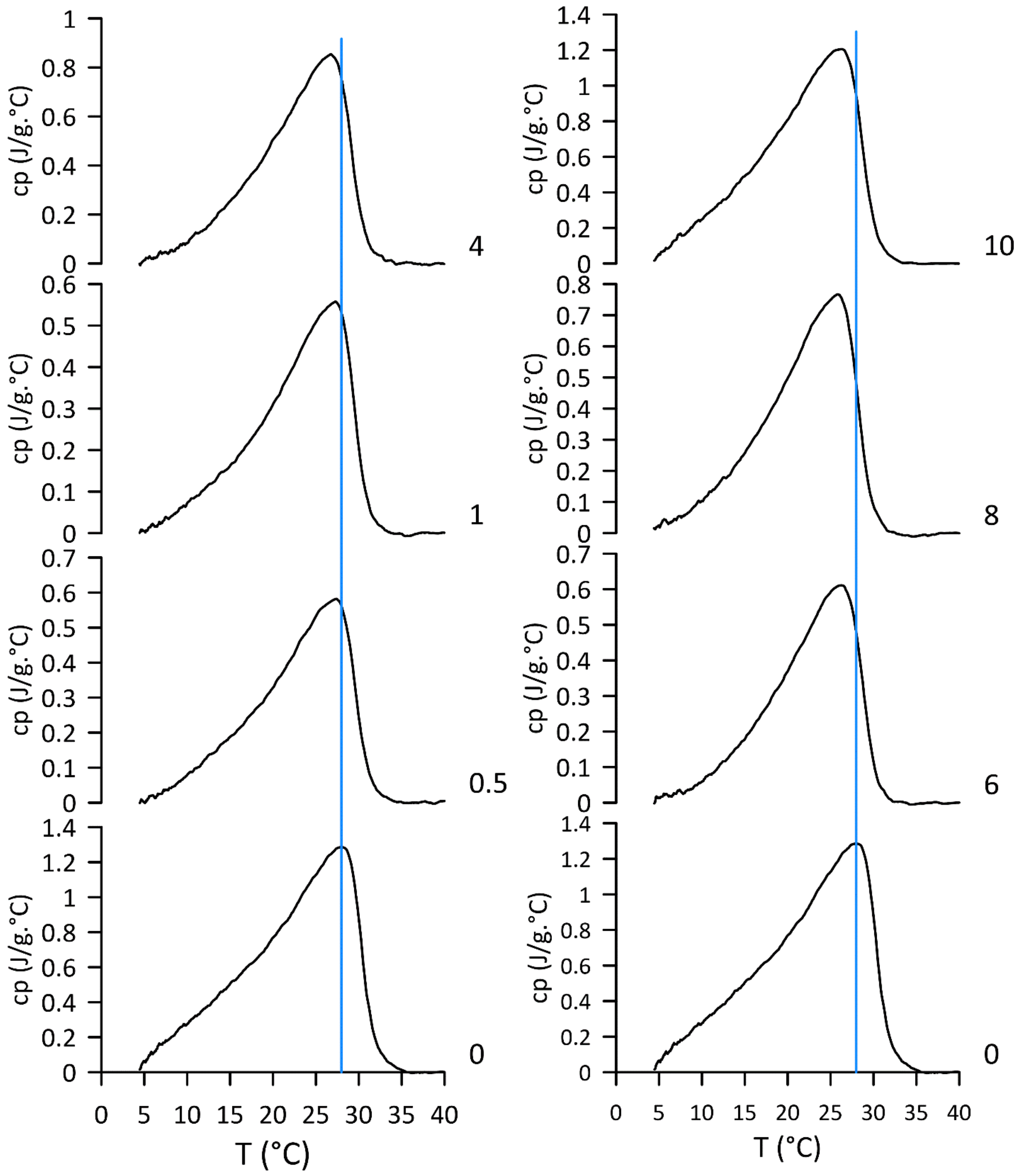


**Figure S2** Individual DSC thermograms of Curosurf® and BUD/Curosurf® mixtures. BUD wt% in each BUD/Curosurf® mixture is presented next to each thermogram. The vertical line indicates the *Tm* of Curosurf®.

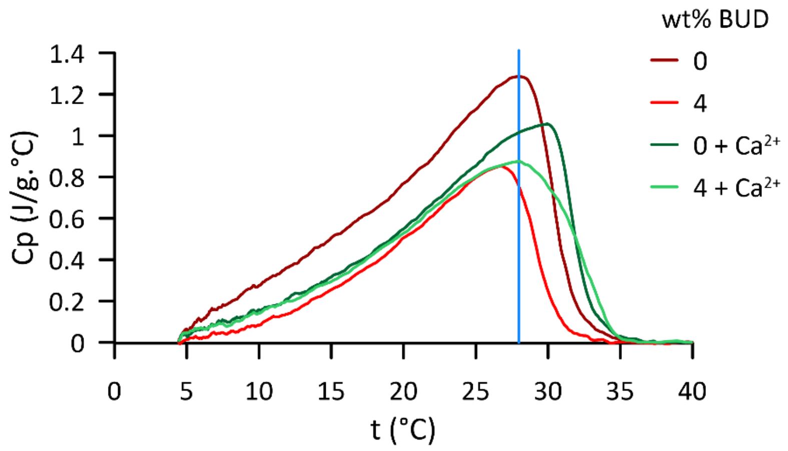


**Figure S3** DSC thermograms of Curosurf® and BUD/Curosurf® for 4 wt% BUD in the presence and absence of 2 mmol/l of $Ca^{2+}$.

**Table S2** DSC derived parameters of Curosurf® and BUD/Curosurf® with 4 wt% BUD in presence and absence of 2 mmol/l of $Ca^{2+}$

| **wt% BUD** | **Tm (°C)** | **ΔH (J/g.°C)** | **ΔH (kcal/mol.°C)*** |
|---|---|---|---|
| 0 + $Ca^{2+}$ | 29.8 ± 0.4 | 14.5 ± 1.0 | 2.6 ± 0.2 |
| 4 + $Ca^{2+}$ | 28.0 ± 0.6 | 9.9 ± 1.0 | 1.8 ± 0.2 |
| 0 | 28.1 ± 0.3 | 18.0 ± 1.5 | 3.2 ± 0.3 |
| 4 | 26.8 ± 0.2 | 7.4 ± 1.0 | 1.3 ± 0.2 |

*Relative molar mass 762 was used [66].

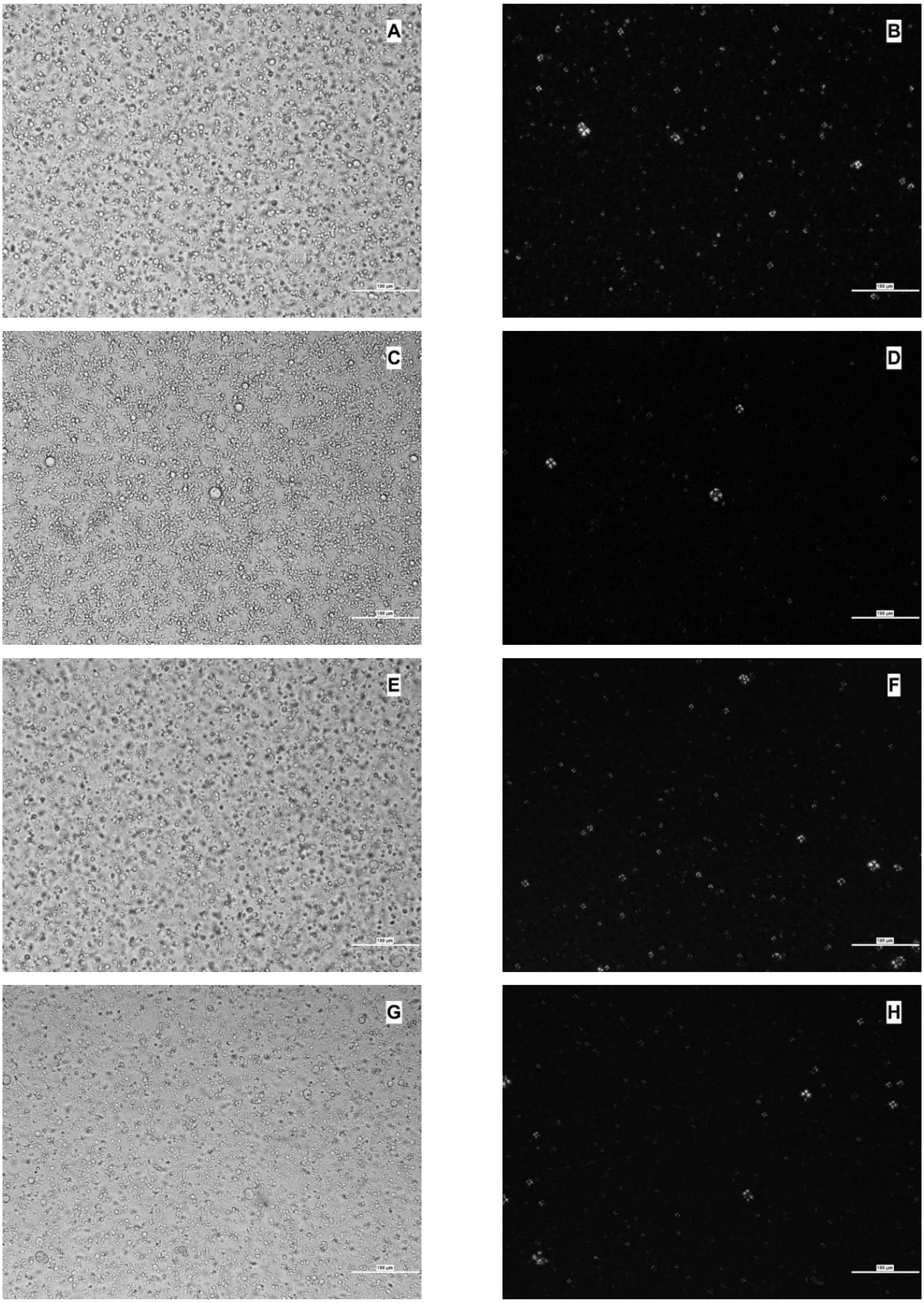


**Figure S4** (A, B) Images recorded in normal (left) and polarized light (right) microscopy for dispersion of Curosurf®; (C, D) BUD/Curosurf® mixture with 4 wt% of BUD; (E, F) Curosurf® mixture in presence of 2 mmol/l of $Ca^{2+}$; (G, H) BUD/Curosurf® mixture with 4 wt% of BUD in presence of 2 mmol/l of $Ca^{2+}$. The scale bars correspond to 100 µm.

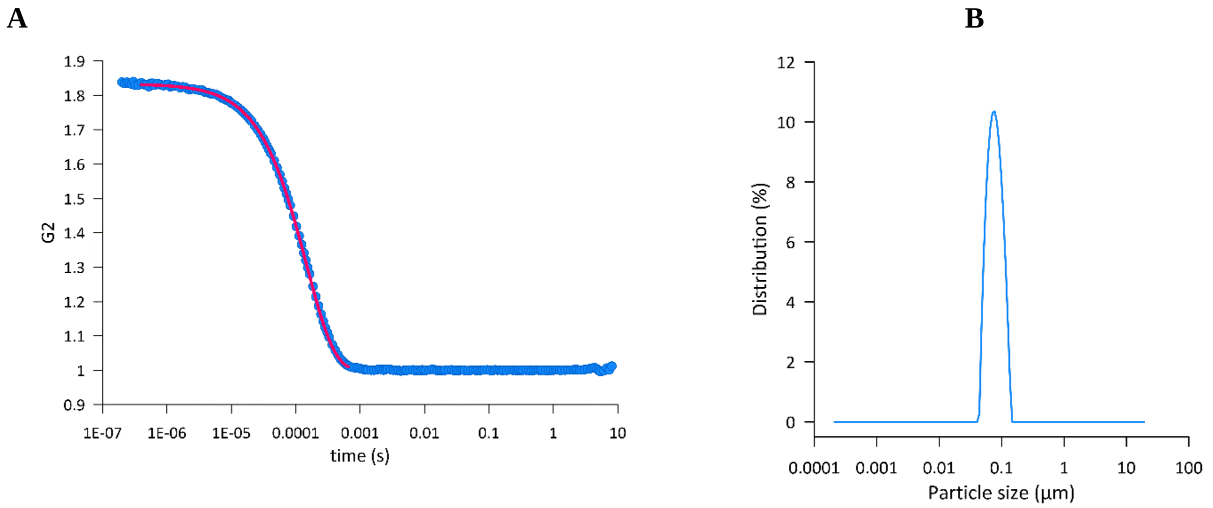


**Figure S5** panel A: time evolution of the correlation function g2 for pure Curosurf ULVs. Exponential decay depicted in red was used to obtain liposome size distribution pictured in panel B.

**Table S3** Comparison of repeat distance *d* of Curosurf® and BUD/Curosurf® at different temperatures.

| | **40 °C** | **45 °C** | **50 °C** |
|---|---|---|---|
| **wt% BUD** | **d (nm)** | **d (nm)** | **d (nm)** |
| 0 (+ $Ca^{2+}$) | 6.9 ± 0.1 | - | 6.9 ± 0.1 |
| 0 | 7.8 ± 0.1 | 7.8 ± 0.1 | 7.6 ± 0.1 |
| 2 | 8.3 ± 0.1 | 8.2 ± 0.1 | 8.1 ± 0.1 |
| 5 | 8.4 ± 0.1 | 8.3 ± 0.2 | 8.2 ± 0.1 |
| 7 | 8.4 ± 0.1 | 8.3 ± 0.1 | 8.2 ± 0.2 |
| 10 | 8.2 ± 0.1 | 8.1 ± 0.1 | 8.0 ± 0.1 |

**Dynamic light scattering (DLS)**

Dynamic light scattering technique was used to study the hydrodynamic diameter and polydispersity of the ULVs in samples prepared for DSC measurement. Backscattering was collected using Litesizer 500 (Anton Paar, Austria) at 20 °C over 60 runs; the duration of each run was 10 s. Measurements were performed in polycarbonate disposable Omega cuvettes (Anton Paar, Austria) with 5 mins equilibration time. Collected experimental data were evaluated using Kalliope software (Anton Paar, Austria). Hydrodynamic diameter $D_h$ is calculated according to the Stokes-Einstein equation:

$$D_h = \frac{k_B T}{3\pi\eta D_t} \qquad \text{(S1)}$$

Where $k_B$ is Boltzmann's constant, T is thermodynamic temperature, $\eta$ is the dynamic viscosity, and $D_t$ is the translational diffusion coefficient.

**A**

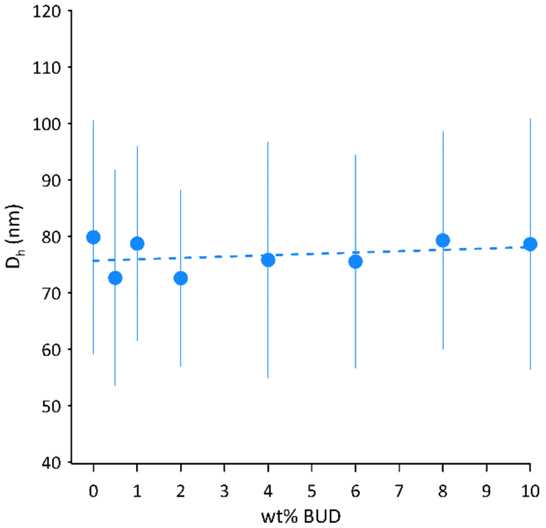


**B**

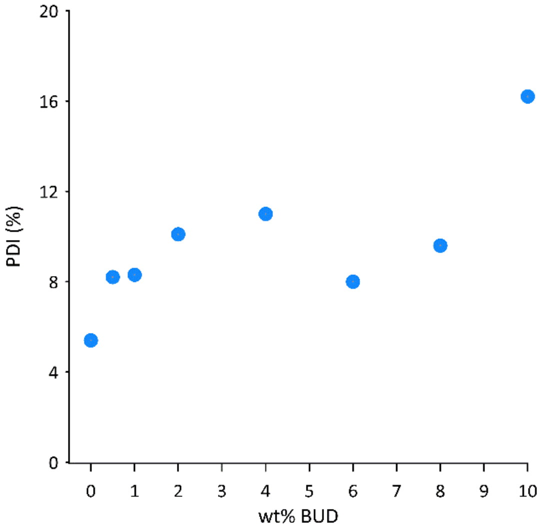


**Figure S6** panel A: The hydrodynamic diameter of liposomes as a function of wt% BUD. The dashed line is an indication of the linear fit of the experimental data. Panel B: polydispersity index as a function of wt% BUD. Error bars are within the size of symbols.

**Electrophoretic light scattering (ELS)**

The electrophoretic light scattering method was used to determine the zeta potential of the Curosurf® ULVs in samples prepared for DSC measurements using Litesizer 500 (Anton Paar, Austria). Unilamellar liposomes prepared for DSC measurement were used for zeta potential measurement. Measurements were performed in polycarbonate disposable Omega cuvettes with volume of 900 μL at 20°C. Zeta potential is calculated according to Henry's equation:

$$\zeta = \frac{3\eta . U_E}{2\varepsilon . f(\kappa a)} \quad \text{(S2)}$$

Where η is dynamic viscosity, $U_E$ is electrophoretic mobility, ε is the dielectric constant, f(κa) is the Henry factor. Kalliope software was used to calculate zeta potential according to the data collected. Reported mean zeta potentials and standard deviations are from three individual measurement each for 60 runs for every sample at 20 °C.

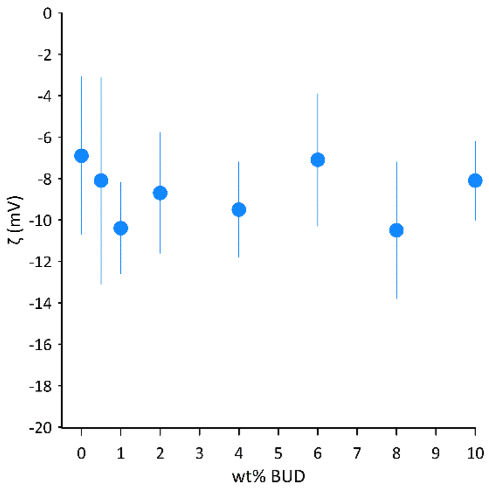


**Figure S7** Mean zeta potential of Curosurf ® ULVs as a function of wt% BUD.

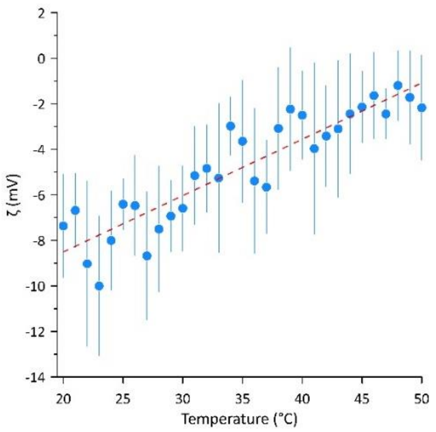


**Figure S8** Zeta potential of Curosurf ® ULVs as a function of temperature. Dashed line is an indication of the linear fit of the experimental data.

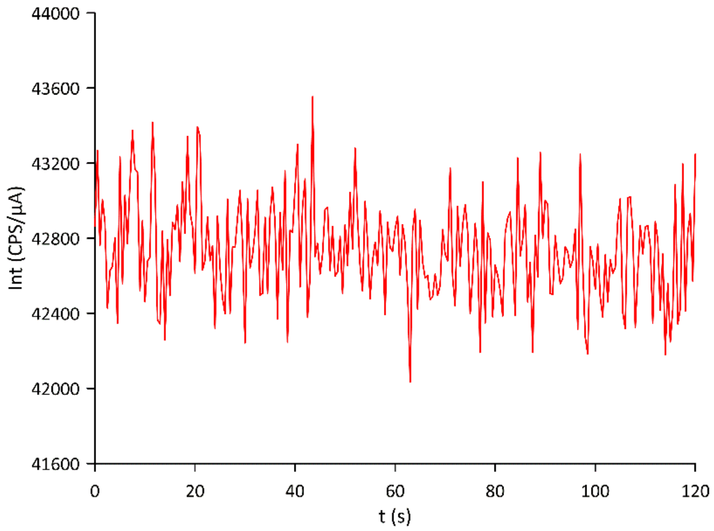


**Figure S9** Time-based fluorescence measurements obtained for the Curosurf® sample at 37 °C. Fluctuation of emission intensity was recorded during 120 s, with $\lambda_{excitation}$ = 376 nm and $\lambda_{emission}$ = 480 nm.

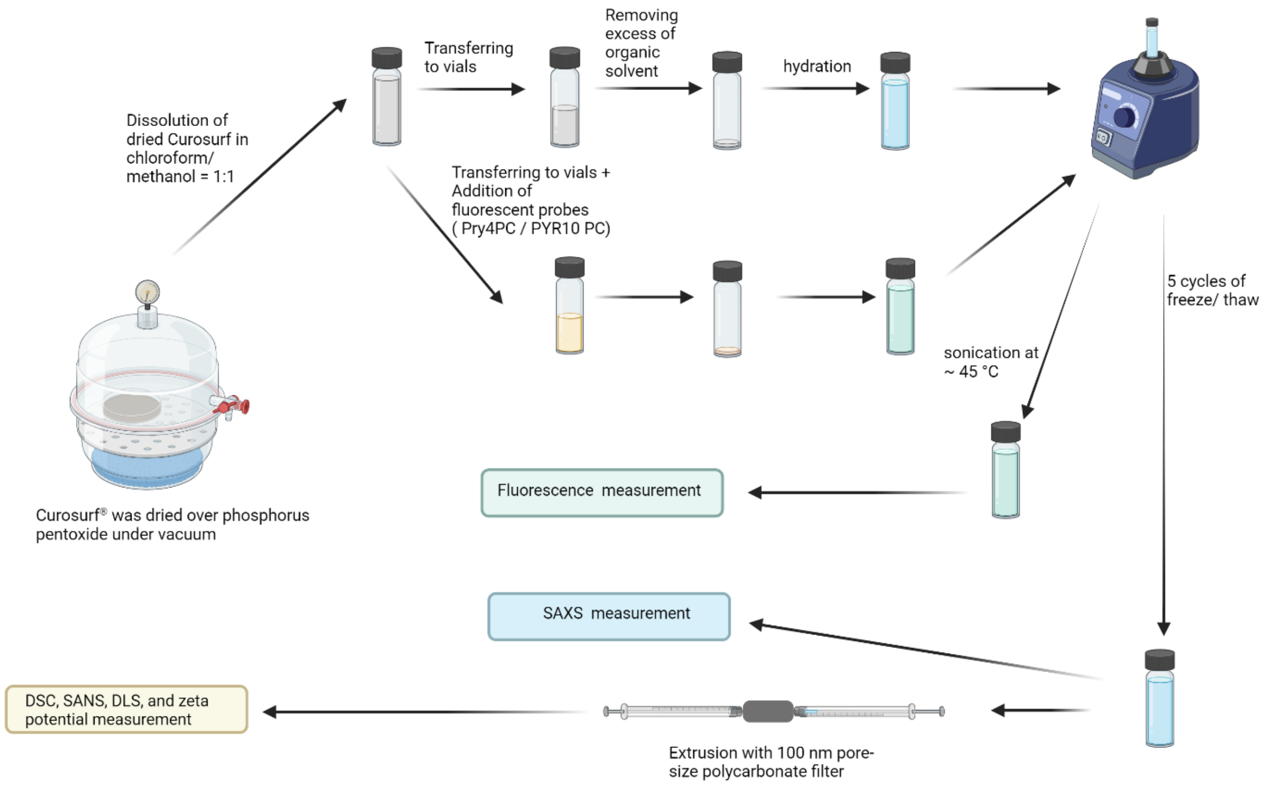


**Scheme S1** Schematic overview of samples preparation.
